# Low-dose exposure of glyphosate-based herbicides disrupt the urine metabolome and its interaction with gut microbiota

**DOI:** 10.1038/s41598-021-82552-2

**Published:** 2021-02-05

**Authors:** Jianzhong Hu, Corina Lesseur, Yu Miao, Fabiana Manservisi, Simona Panzacchi, Daniele Mandrioli, Fiorella Belpoggi, Jia Chen, Lauren Petrick

**Affiliations:** 1 Department of Genetics and Genomic Sciences, Icahn School of Medicine at Mount Sinai, 1425 Madison Avenue, New York, NY USA; 2 Department of Environmental Medicine and Public Health, Icahn School of Medicine at Mount Sinai, 1425 Madison Avenue, New York, NY USA; 3 Cesare Maltoni Cancer Research Center (CMCRC), Ramazzini Institute (RI), Bentivoglio, Bologna, Italy; 4 Department of Veterinary Medical Sciences, University of Bologna, Bologna, Italy; 5 Department of Agricultural Sciences, University of Bologna, Bologna, Italy; 6 Institute for Exposomics Research, Icahn School of Medicine at Mount Sinai, New York, NY USA

**Keywords:** Environmental impact, Biomarkers

## Abstract

Glyphosate-based herbicides (GBHs) can disrupt the host microbiota and influence human health. In this study, we explored the potential effects of GBHs on urinary metabolites and their interactions with gut microbiome using a rodent model. Glyphosate and Roundup (equal molar for glyphosate) were administered at the USA glyphosate ADI guideline (1.75 mg/kg bw/day) to the dams and their pups. The urine metabolites were profiled using non-targeted liquid chromatography—high resolution mass spectrometry (LC-HRMS). Our results found that overall urine metabolite profiles significantly differed between dams and pups and between female and male pups. Specifically, we identified a significant increase of homocysteine, a known risk factor of cardiovascular disease in both Roundup and glyphosate exposed pups, but in males only. Correlation network analysis between gut microbiome and urine metabolome pointed to *Prevotella* to be negatively correlated with the level of homocysteine. Our study provides initial evidence that exposures to commonly used GBH, at a currently acceptable human exposure dose, is capable of modifying urine metabolites in both rat adults and pups. The link between *Prevotella*-homocysteine suggests the potential role of GBHs in modifying the susceptibility of homocysteine, which is a metabolite that has been dysregulated in related diseases like cardiovascular disease or inflammation, through commensal microbiome.

## Introduction

Glyphosate-based herbicides (GBHs), such as Roundup, are currently the most widely used herbicides in the world. GBHs are complex proprietary mixtures, with glyphosate as the main active ingredient. Since glyphosate was first produced in 1974 approximately 9.4 million tons of GBHs have been sprayed globally, nearly half a pound of glyphosate on every cultivated acre of land^1^. After the introduction of genetically modified organisms (GMOs) in 1996, in particular, glyphosate resistant crops, the global usage of GBHs has been increasing exponentially with about two-thirds of the total GBHs (by mass) sprayed in just the last decade. Besides GMO application, GBHs are also applied on non-GMO crops as desiccant in order to dry down crops and accelerate the harvest^2^. This practice further increases dietary exposure to glyphosate and its major metabolite aminomethylphosphonic acid (AMPA)^3^. Glyphosate mode of action is the inhibition of 5-enolpyruvylshikimate-3-phosphate synthase (EPSPS), involved in the synthesis of three aromatic amino acids: tyrosine, tryptophan, and phenylalanine^4^. As this shikimate pathway only exists in bacteria, fungi, and plants, but not in vertebrates, glyphosate was thought to impose minimal risks to mammals including humans. However, current emerging evidence suggests that glyphosate or GBH such as Roundup, can adversely affect mammalian biology via multiple mechanisms^5–8^. Several studies have also suggested the possible link between GBHs exposure and abnormality in neurodevelopment^9,10^. Among those mechanisms, it has been reported that GBHs exposure can alter the microbiota in honeybees, rats and other animals^11,12^ and recently a shotgun metagenomic approach revealed that glyphosate alters the gut microbiome of Sprague–Dawley (SD) rats by inhibiting the shikimate pathway^13^. The gut microbiome is known to be a key player in modulating host metabolism; with multiple studies describing strong correlations between gut microbiome (microbial composition and metabolic function) and host metabolites^14–17^. Several microbial species and microbial metabolic functions have also been suggested to play a leading role in gut-systemic metabolic interplays^16,18^. Therefore, we speculate that GBHs exposure may have the potential to modify the human microbiota, and which, in turn, to influence host metabolic functions.

Metabolomics is a promising approach to study the associations between environmental exposures and health effects^19,20^. Application of metabolomics profiling can potentially yield novel biomarkers, and inform molecular alterations underlying the toxicities. Over the last few years, many toxicological studies have demonstrated that metabolomics is a highly sensitive method in detecting effects associated with environmental exposures, where metabolite perturbations often happened prior to the histopathological changes^18,21,22^. However, to date, few studies have been carried out on the effect of glyphosate exposure on host metabolomics profiles. In particular, urinary metabolomics has not been used to investigate the biological perturbations of glyphosate exposure and interactions between GBH altered microbiome during early life, a critical period of susceptibility as denoted in the Developmental Origins of Health and Disease (DOHaD) paradigm. Therefore, in this pilot study, we performed untargeted metabolomics profiling in Sprague–Dawley (SD) rats to examine whether exposure to GBH at low doses comparable to those ubiquitously exposed to humans, can affect urinary metabolomics profiles during early development. The animals in this study have been previously investigated for microbiome changes by glyphosate or Roundup exposures^11^, providing initial evidence that exposures to commonly used GBHs, at doses considered safe, are capable of modifying the gut microbiota in early development, particularly before the onset of puberty. Combining with existing gut microbiome data, we further test whether the GBH altered metabolites in paired urine correlate with changes in the gut microbiome.

## Methods

### Animals

The animal study was carried out in compliance with the **ARRIVE** (Animal Research: Reporting of In Vivo Experiments) guidelines. All animal study procedures were performed at the Cesare Maltoni Cancer Research Centre/Ramazzini Institute (CMCRC/RI) (Bentivoglio, Italy), where the animal experiments were conducted with strict adherence to the Italian law regulating the use and treatment of animals for scientific purposes (Decreto legislativo N. 26, 2014. Attuazione della direttiva n. 2010/63/UE in materia di protezione degli animali utilizzati a fini scientifici.—G.U. Serie Generale, n. 61 del 14 Marzo 2014). Before starting, the protocol was examined by the Internal Ethical Committee for approval. The protocol of the experiment was also approved and formally authorized by the ad hoc commission of the Italian Ministry of Health (ministerial approval n. 710/ 2015-PR). As previously described^23^, female breeders SD rats were placed individually with a single unrelated male until evidence of copulation was observed. After mating, females were housed separately during gestation and delivery. Newborns were housed with their mothers until weaning. Up to 2 pups per litter were randomly selected for the study in order to have minimal differences in body weight among the treatment groups, with a standard deviation of no more than 10% from the average. This ensured comparable dosing of glyphosate or Roundup though drinking water. The Weaned pups were housed by treatment group and sex in Makrolon cages (cm 41 × 25 × 15) at two or three pups per cage. The metabolic cages had a stainless-steel wire top and a shallow layer of white firewood shavings as bedding. All animals were kept in a single room at 23 ± 3 °C and at 40–60% relative humidity with light/dark cycles at 12 h each using artificial light. The animals were given the same standard “Corticella” pellet diet (Piccioni Laboratory, Milan, Italy) for both breeders and offspring; both feed and tap water were available ad libitum. Feed and tap water were routinely analyzed to exclude biological and chemical contamination (mycotoxins, pesticides, arsenic, lead, mercury, selenium).

### Treatment

**Figure 1:**
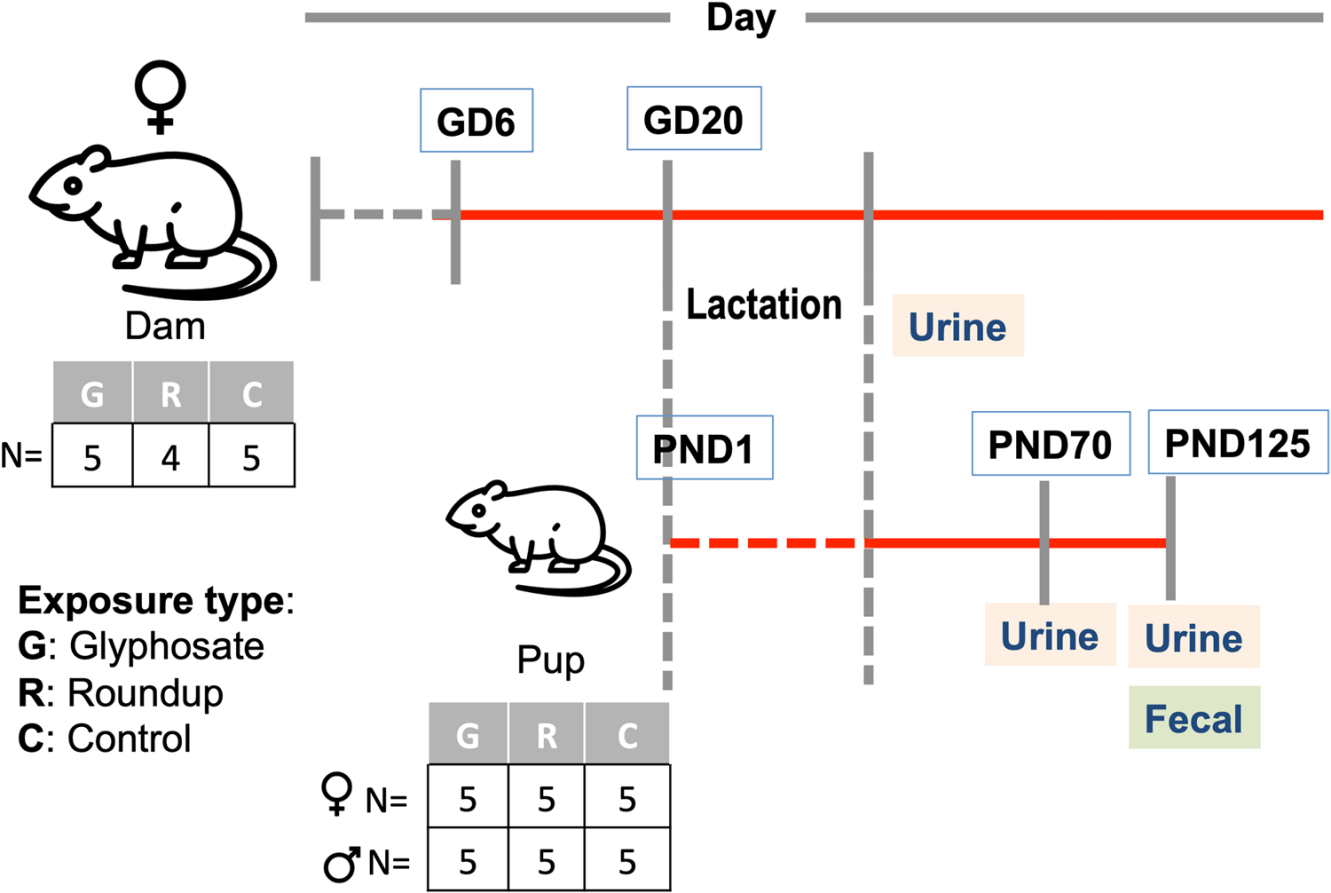
Study design. Test compounds including pure glyphosate and Roundup are administered ad libitum in drinking water. Fourteen dams are treated through drinking water starting from the gestation day 6th (GD 6). Thirty pups are treated from embryonic life (GD 6) indirectly from dams milk following birth (GD20) until the end of lactation, then directly through drinking water for 90 days after weaning (until PND 125). Urine samples are collected after lactation from dams and PND70 and PND 125 from pups. Fecal samples are collected at PND 125 from pups.

The timeline of the experimental animal treatment and sample collection has been described previously^11,23^. As illustrated in Fig. 1, we selected 14 dams (N = 5 controls, N = 5 glyphosate and N = 4 Roundup) and 30 F1 pups (15 female and 15 male). The F0 dams received the treatment through drinking water from gestation day (GD) 6 to the end of lactation (totally they were exposed for 49 ± 2 days). The F1 pups received the treatment from their dams starting from in utero (GD 6) and mainly through milk during lactation. After weaning, F1 pups were treated through drinking water until sacrifice (PND 70 or PND 125).

### Urine and fecal sample collection

Urine samples were collected using a metabolic cage, specifically designed for separate collection of urine and feces. Urine samples were collected at the end of lactation for dams and at PND70 and PND125 for pups. The urine samples were centrifuged to remove any debris (50,000 × g at 10 °C for 15 min) then transferred to 1.5 ml cryovials. Fecal samples (2–3 droppings) were freshly collected from the anus of each animal, limiting potential contamination. About 2–3 fecal droppings from each pup were collected as described previously^11^. Briefly, forceps used for collecting droppings were washed and cleaned using sterile water and 1% sodium bicarbonate between each sampling to avoid cross contamination. The urine and fecal cryovials were stored at − 20 °C until shipment on dry ice to the testing laboratories at Icahn School of Medicine at Mount Sinai.

### Metabolomics analysis

Urine samples were thawed on ice, vortexed and diluted with water down to a specific gravity of 1.002 for pre-acquisition normalization^24^. A 20 µL aliquot of the diluted sample was prepared and stored at − 80 °C until metabolomics analysis. Immediately prior to liquid chromatography—high resolution mass spectrometry (LC-HRMS) analysis, urine samples were combined with 180 µL of acetonitrile, containing internal standards, to precipitate proteins. The supernatant was transferred to LC vials.

Sample extracts were analyzed in ZIC HILIC positive (ZHP) and RP negative (RPN) modes separately using established methods^25^. Samples were maintained at 5 °C in the autosampler module. For polar metabolites separation, 2 µL of sample was injected onto a HILIC SeQuant ZIC-HILIC column (100 mm × 2.1 mm, 100 Å, 3.5 µm particle size, Merck, Darmstadt, Germany) maintained at 25 °C. While for nonpolar metabolites separation, 2 µL of sample sandwiched between 10 µL of water was injected onto a Zorbax Eclipse Plus C18, RRHD column (50 mm × 2.1 mm, 1.8 µm particle size, Agilent Technologies, Santa Clara, USA) coupled to a guard column (5 mm × 2 mm, 1.8 µm Agilent Technologies, Santa Clara, USA) maintained at 50 °C. Samples were analyzed in randomized order**.** To monitor system stability, a pooled QC sample prepared by combining aliquots of all samples was injected routinely throughout the run.

### LC–MS pre-processing

Suspect screening was performed using an in-house database of over 600 authentic standards analyzed under the same conditions. For untargeted data analysis, the raw data files were first converted into mzxml^26^ format and a peak table generated using [XCMS]^27^ with parameters optimized by [IPO]^28^. Metabolite features with a CV < 30% in the pooled-QC injections and with a mean fold change > 3 or > 1.5 compared to blank extracts for untargeted analysis or suspect screening, respectively, were retained for further analysis. The metabolomics data was further imputed by k-nearest neighbor imputation using [knn_impute] with a cutoff = 0.4 (40% missing values) and normalized by [normalize_met] with default settings using the R package [MetaboDiff]^29^. After imputation, 3 out of 154 metabolites were removed due to > 40% missing values.

### Statistical analysis

Differential metabolomic analysis was performed using R package [MetaboDiff]^29^. Unsupervised principal component analysis (PCA) was performed to compare overall metabolomic profiles by age, sex and exposure types. Tests for statistical significance were performed with the PERMANOVA test (adonis function in the vegan R package)^30^. Supervised partial least squares discriminant analysis (PLS-DA) was performed using the mixOmics R package^31^ to select the major contributing metabolomic features that differentiate between study groups. A PLS-DA Variable Importance in Projection (VIP) score > 2.0 was used as the cutoff value to identify top features contributing to metabolic differences. For each selected feature, we also compared the mean by non-parametric two-sample Mann–Whitney test and *p* values were FDR adjusted for multiple comparisons. Alternatively, we used the random forests (RF) algorithm, a supervised machine learning approach, using R package [Boruta]^32^ to identify significant differential metabolites associated with exposures. The microbiome data of pups at PND125 was obtained from our previous study^11^. Briefly, the rat gut microbiome was surveyed using 16S rRNA gene sequencing on the phylogenetically informative V3–V4 region as previously described^33^. The QIIME 2.0 pipeline^34^ combined with DADA2^35^ was used to process the sequencing data. The microbiome composition was further analyzed using R package [phyloseq]^36^. Correlation analysis was performed to investigate relationships between selected metabolites and gut microbiota. For each microbiota and metabolite with FDR adjusted *p* value < 0.05, an edge was added between the corresponding nodes and visualized as a network with the edge color as the direction of the correlation and the edge width as the absolute values of the log10 (FDR adjusted *p* values). The spearman correlation networks between microbiome and metabolites were constructed using R package [igraph]^37^ and FDR adjusted *p* values were obtained using R package [qvalue]^38^.

### Ethical approval and consent to participate

The animal protocol was examined by the Internal Ethical Committee for approval. The protocol of the experiment was also approved and formally authorized by the ad hoc commission of the Italian Ministry of Health (ministerial Approval No. 710/ 2015-PR).

## Results

### Metabolomic profile between sex and adulthood groups

**Figure 2:**
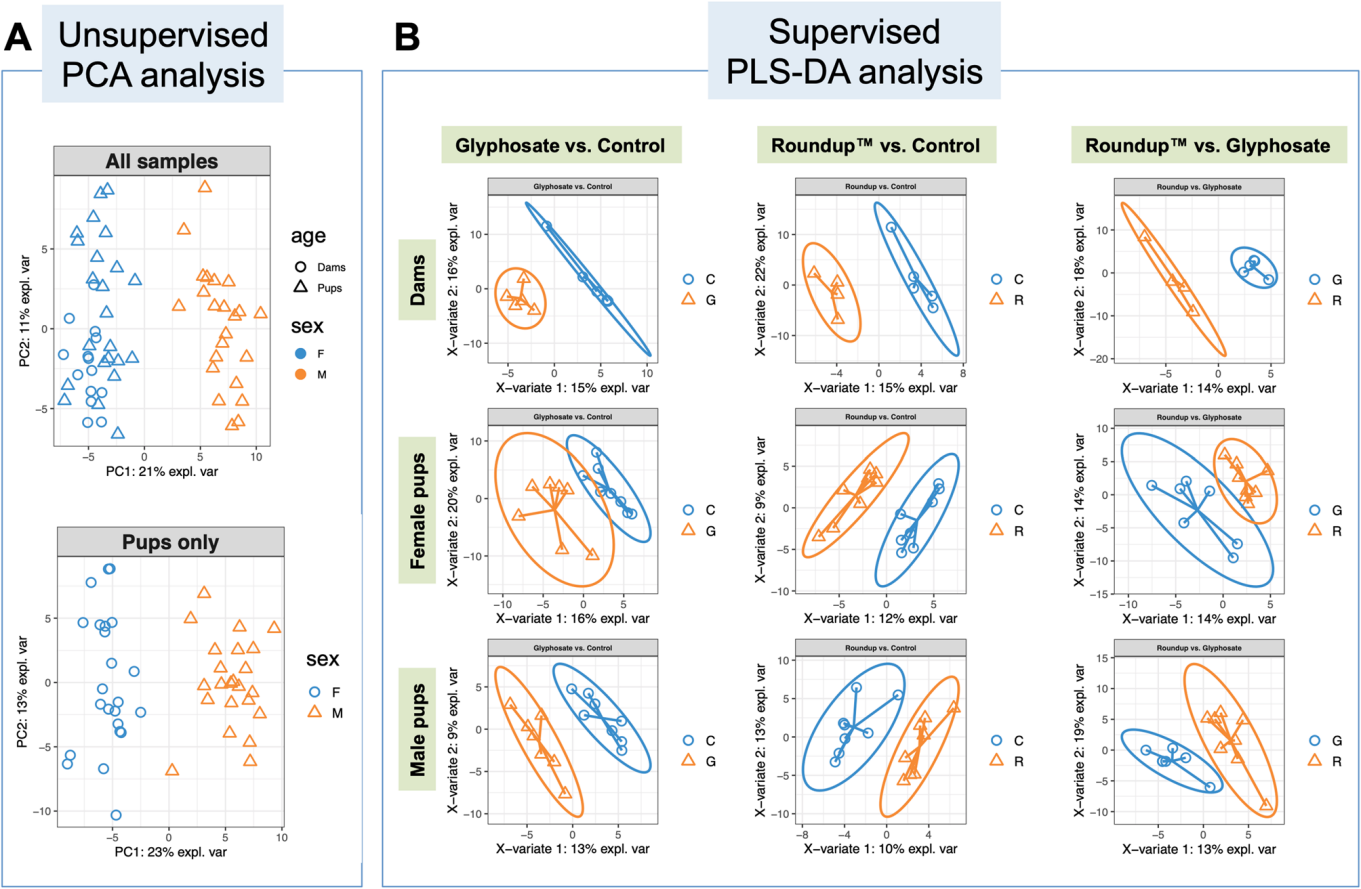
Differential metabolomic profiles by glyphosate exposure in rat female and male pups. (**A**) Unsupervised PCA analysis shows the metabolomic profiles were differentiated by adulthood and sex. (**B**) Supervised Partial Least Squares Discriminant Analysis (PLS-DA) shows distinct metabolome clusters that are strongly associated with the exposure chemicals among groups by adulthood and sex.

After filtering, untargeted metabolomic profiling resulted in 4637 peaks in RPN and 5346 peaks in ZHP for further statistical analysis. An unsupervised principal components analysis was initially employed to compare the overall metabolomic profiles and detect outliers. We found the overall metabolomic profiles differed significantly between dams and pups, and between male and female pups, but not by exposure group (glyphosate, Roundup or control) (*P* values = 0.001, 0.001 and 0.17, respectively by multivariate PERMANOVA test, Fig. 2A and Supplementary Fig. S1).

### Metabolomic features by exposure group and sex

For the discrimination of the exposure types, the Partial Least Squares Discriminant Analysis (PLS-DA), a supervised clustering method was used (Fig. 2B) using the 151 metabolites identified through suspect screening. We used the function perf to evaluate the PLS-DA model using fivefold cross-validation repeated 50 times and decided the optimal number of components = 3 based on the error rate changes (Fig. S2). Metabolites identified in sets with different exposure groups (Glyphosate, Roundup or non-exposed control) in dams and female or male pups were selected by using the cutoff of PLS-DA Variable Importance in Projection (VIP) score > 2.0 and a *p* value < 0.05 in the metabolite fold change level. The full list of metabolites with VIP scores, median expression values and the interquartile ranges are listed in Tables S1–S3, while the selected metabolites are shown in Tables 1, 2, 3 and Fig. S3. Distinct differential metabolites by exposure types were found in both dams and pups. In dams (Table 1, Fig. S4), glyphosate-exposed animals had significantly reduced methionine levels compared to controls. Glyphosate-exposed dams also showed significant reduced 2-methylglutarate, dimethylglycine and beta-alanine methyl ester and increased pipecolate and riboflavin compared to Roundup. In female pups (Table 2 and Fig. S4), sebacic acid, suberate, 10-hydroxydecanoate and adenine were significantly increased while n-methylglutamate and aminocaproate were reduced in glyphosate-exposed compared to controls; whereas adenine was significantly increased in Roundup exposed animals compared to controls. Of note, there was a trend of increased urinary adenine in both glyphosate and Roundup exposed female pups. However, the VIP score was > 2 only in the Roundup versus control comparison. In male pups (Table 3 and Fig. S4), glyphosate exposure resulted in significantly increased 1-aminocyclopropanecarboxylate and homocysteine and significantly reduced mevalolactone compared to controls; whereas Roundup exposure resulted in significantly increased homocysteine and significantly reduced phenylethanolamine, 2-oxobutanoate and biopterin compared to controls. Importantly, compared to non-exposed male controls, both glyphosate and Roundup exposed animals displayed a significant increase in urinary homocysteine levels. We did not observe overlapping significant features shared between dams and pups or between female and male pups.

**Table 1:** The VIP score and fold change of metabolites significantly differentially measured between exposure groups in dams.

Metabolites*	Glyphosate vs. Control			Roundup™ vs. Control			Glyphosate vs. Roundup™		
	VIP score	P	P-adj**	VIP score	P	P-adj**	VIP score	P	P-adj**
**L-Methionine**	2.43	0.0079	**0.048**	–	0.11	0.38	–	> 0.99	> 0.99
2-Methylglutarate	–	0.22	0.67	–	> 0.99	> 0.99	2.38	0.016	**0.038**
Pipecolate	–	0.69	> 0.99	–	0.56	0.84	2.30	0.032	**0.038**
Riboflavin	–	0.55	> 0.99	–	0.73	0.88	2.26	0.032	**0.038**
Dimethylglycine	–	> 0.99	> 0.99	–	0.19	0.38	2.24	0.032	**0.038**
Beta-alanine methyl ester	–	> 0.99	> 0.99	–	0.19	0.38	2.22	0.032	**0.038**

*VIP score = Variable Importance in Projection scores obtained from PLS-DA analysis. Differences in mean values were assessed by two-sample Mann–Whitney test. Metabolites in bold were differential features confirmed by random forest feature selection method.

**FDR-adjusted *p* values.

**Table 2:** The VIP score and fold change of metabolites significantly differentially measured between exposure groups in female pups.

Metabolites*	Glyphosate vs. Control			Roundup™ vs. Control			Glyphosate vs. Roundup™		
	VIP score	P	P-adj**	VIP score	P	P-adj**	VIP score	P	P-adj**
**Sebacic acid**	2.56	0.0059	**0.022**	–	0.1	0.17	–	0.54	0.79
**N-Methylglutamate**	2.39	0.0093	**0.022**	–	0.065	0.17	–	0.87	0.87
**Suberate**	2.29	0.0093	**0.022**	–	0.16	0.21	–	0.61	0.79
10-Hydroxydecanoate	2.14	0.029	**0.039**	–	0.19	0.22	–	0.46	0.79
Aminocaproate	2.06	0.014	**0.022**	–	0.1	0.17	–	0.54	0.79
**Adenine**	–	0.014	**0.022**	2.29	0.0011	**0.0087**	–	0.69	0.79

**Table 3:** The VIP score and fold change of metabolites significantly differentially measured between exposure groups in male pups.

Metabolites*	Glyphosate vs. Control			Roundup™ vs. Control			Glyphosate vs. Roundup™		
	VIP score	P	P-adj**	VIP score	P	P-adj**	VIP score	P	P-adj**
**1-Aminocyclopropanecarboxylate**	2.37	0.0093	**0.033**	–	0.082	0.19	–	> 0.99	> 0.99
**Homocysteine**	2.30	0.0059	**0.033**	2.49	0.01	**0.036**	–	0.87	0.93
**Mevalolactone**	2.29	0.0093	**0.033**	–	0.79	0.8	–	0.23	0.30
**Phenylethanolamine**	–	0.15	0.19	2.74	0.0029	**0.033**	–	0.072	0.11
**2-Oxobutanoate**	–	0.04	0.08	2.55	0.015	**0.041**	–	0.19	0.26
**Biopterin**	–	0.15	0.19	2.33	0.0047	**0.033**	–	0.054	0.095

In addition to PLS-DA, we also performed the metabolomic feature selection using RF machine learning feature selection method with the Boruta algorithm. Among the metabolites selected by RF (Table S2, Fig. S5), we found many selected features were consistent with the results from the PLS-DA.

### Correlations between metabolomics and microbiota

**Figure 3:**
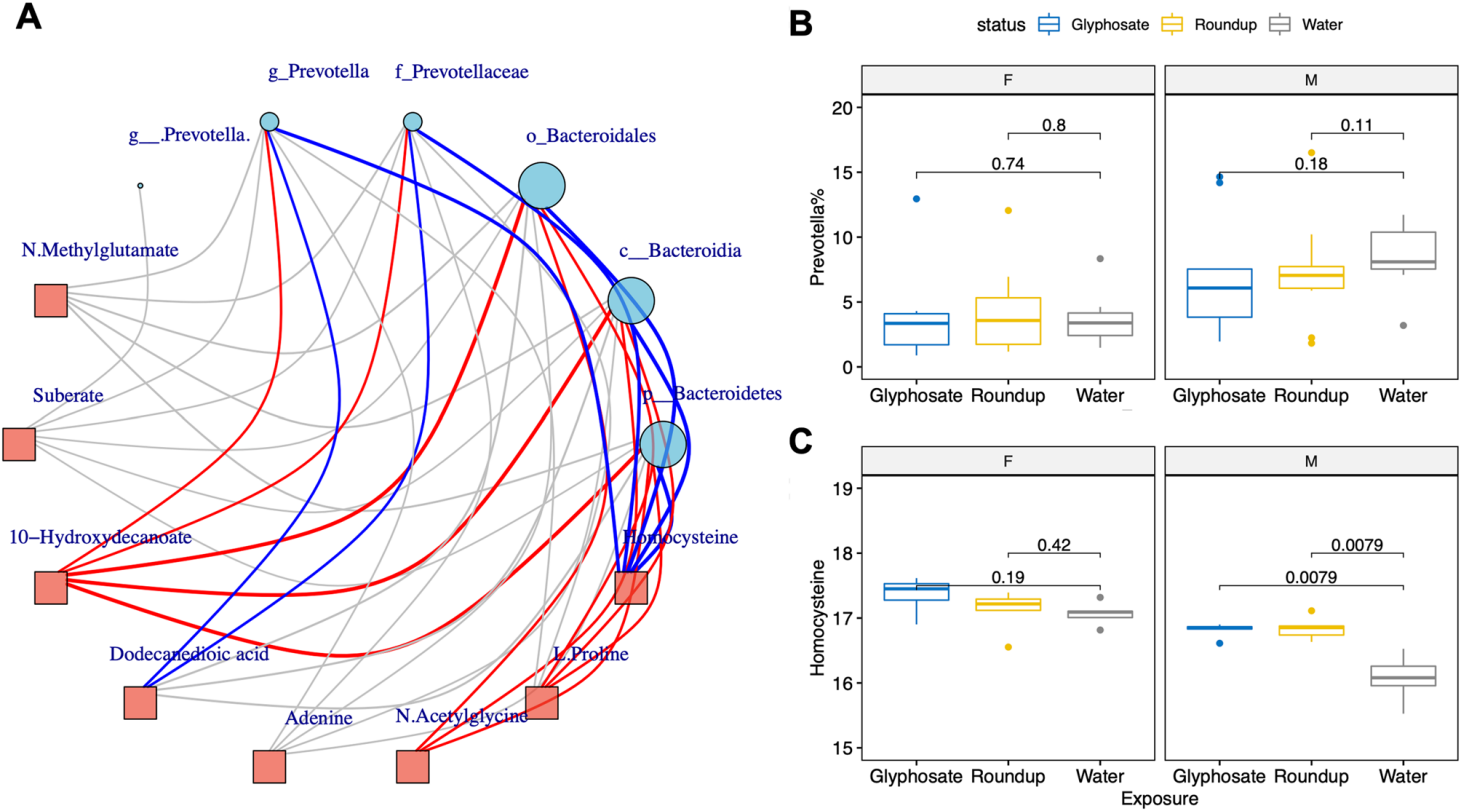
The urine homocysteine levels in pups are associated with sex and glyphosate or Roundup exposure and are strongly correlated with *Prevotella* abundance in gut microbiota. (**A**) Correlation network between exposure associated metabolic features and gut microbiota. *Prevotella* and its belonged *Bacteroidetes* phylum to *Prevotellaceae* family all show strong negative correlation with the urine homocysteine. The links with FDR adjusted *p* value < 0.05 are colored with red (positive correlation) and blue (negative correlation). (**B**,**C**) Boxplots showed that *Prevotella* abundances were lower in female pups than male pups. *Prevotella* was reduced in exposed male pups. In contrast, the female pups have higher homocysteine than the male pups and the homocysteine levels were significantly increased by exposure in male pups.

To test whether top metabolites selected by PLS-DA in this study are linked to gut microbiota, we performed a correlation-based network analysis between differential metabolites (VIP > 2) in pups at PND125 and paired gut microbial composition from the same animal at PND125. Overall results from the metabolite-microbial correlation analyses are presented in Fig. 3A. We found the *Prevotella* genus was strongly correlated with 10-hydroxydecanoate (rho = 0.57, FDR adjusted *p* value = 0.015), dodecanedioic acid (rho = − 0.58, FDR adjusted *p* value = 0.012) and homocysteine (rho = − 0.64, FDR adjusted *p* value = 0.0028). It should be noted that urinary homocysteine levels are also negatively correlated with not only *Prevotella* genus, but also with its phylum, class, order and family. Similarly, the level of 10-hydroxydecanoate is positively correlated with not only *Prevotella* genus but also its taxonomic hierarchy *Bacteroidetes* phylum, *Bacteroidia* class, *Bacteroidales* order and *Prevotellaceae* family. In sex-stratified analyses (Fig. 3B,C), male pups showed a clear inverse relationship between the levels of homocysteine and relative abundance of *Prevotella*. Female pups showed a similar trend; however, the changes did not reach statistical significance. Since *Prevotella* has been correlated with microbial alpha-diversity^39^, we conducted additional regression analysis and found that the abundance of *Prevotella* is significantly associated with the level of homocysteine with or without adjustment to alpha-diversity Shannon index (*p* value = 0.0024, or 0.0085, respectively).

## Discussion

GBHs are the most applied herbicides worldwide and humans are commonly exposed to these environmental chemicals at various doses. Environmental GBHs are ubiquitous and GBHs residues can be found in food^40^, drinking-water^41^, crops^42^, animal feed^43^, groundwater^3^, rain^44^ and even in air^45^. Although the effects of GBHs on human health are under intense public debate, evidence is emerging that they impact many health outcomes, including developmental and reproductive toxicity^46–48^, endocrine disruption^49,50^, host immunity^51–53^, obesity and diabetes^7,54^, gastrointestinal disorders ^55^, cardiovascular disorders ^56,57^ and central nervous system dysfunction such as learning and memory impairment^58^, anxiety, depression^59^ and autism^8^. These chronic health outcomes may occur even at doses lower than established risk safety guidelines, in particular during critical development windows as denoted in the DOHaD paradigm^60^. Environmental exposures may lead to changes in metabolism^20,61^. Comprehensive, unbiased metabolite profiling using untargeted metabolomics is a promising approach to study the associations between environmental exposures and health effects. Although our sample size was small, our results showed that gestational and early-life low-dose exposure to glyphosate or Roundup significantly altered multiple urine metabolomic biomarkers, in both dams and offspring.

The one-carbon metabolism is a metabolic process that serves to activate and transfer 1C units for biosynthetic processes including purine and thymidine synthesis and homocysteine remethylation^62^. Folate is the essential cofactor in the one-carbon cycle. Animals and humans cannot biosynthesize folate, thus requiring dietary intake or absorption of folate biosynthesized by gut microbiota^63^. In this study, we observed that low-dose GBH exposure can influence multiple metabolites involved in one-carbon metabolism. One key metabolite induced by GBHs exposure in male pups is homocysteine, a non-proteinogenic α-amino acid, biosynthesized from methionine that can be remethylated back into methionine or converted into cysteine with the aid of certain B-vitamins. Homocysteine metabolism is highly dependent on vitamin derived cofactors; deficiencies in vitamin B12, folic acid and vitamin B6 are associated with higher levels of homocysteine in blood (hyperhomocysteinemia). In addition to homocysteine, we also observed GBH-induced changes in methionine and N-methylglutamate also involved in one carbon metabolism. Interestingly, probiotic bacteria, including *Prevotella* using products of the shikimate pathway, which is inhibited by GBHs, can biosynthesize B vitamins including folate^64^. Thus, it is plausible that the increased urine homocysteine we observed in male pups exposed to low-dosage GBHs results from reduced production of folic acid by *Prevotella* bacteria, paralleling the increase in homocysteine in dietary vitamin deficiencies.

Although the potential mechanism is still not clear, studies found that children with autism spectrum disorder (ASD) lack microbial diversity and have a decreased abundance in probiotics including *Prevotella*^65,66^, potentially leading to reduced folate production by microbiota in individuals with ASD^67^. As ASD as well as brain damage, cognitive and memory decline have been linked with higher levels of homocysteine^68–72^, we hypothesize that induced homocysteine by environmental exposure to GBHs during early life may contribute to the development of ASD or other neurodevelopmental disorders.

To be noted is that distinct metabolites were not only found between exposed and non-exposed, but also found between glyphosate and Roundup exposed animals. Previous experimental evidence^73–75^ supports that the glyphosate formulations like Roundup are more toxic than glyphosate alone; however, the underlying mechanisms are still not clear. Our results suggested that metabolite profiling might be useful to identify possible metabolic pathways and to explain the excessive toxicity in those formulations.

This study has its limitations, mainly due to its small sample size, thus statistical power was limited in subgroup analyses. Secondly, glyphosate and its hydroxylated metabolites could not be detected in the metabolomics method. Furthermore, the microbial survey using 16S rRNA gene amplicons-sequencing techniques in our study cannot capture the full metabolic activity of the microbial features correlated with host metabolomics. A more comprehensive whole genome metagenomic sequencing approach may be required for a full spectrum microbial metabolic function profiling to find underlying mechanistic links between gut microbiome and the host metabolism.

In conclusion, to our knowledge, this is the first study on GBH-induced urinary metabolomic changes at doses currently considered safe in humans. Metabolomic analyses revealed differences in urinary metabolite levels associated with GBH exposure. The link between *Prevotella*-homocysteine suggests a potential role of commensal microbiome in modulating the metabolic alteration by GBH exposure. Limitations of this study include sample size and analysis of only a single urine sample. Therefore, further studies should include a larger sample size, especially for subgroup analysis, as well as longitudinal samples to investigate underlying mechanistic associations and windows of susceptibility more comprehensively.

## Supplementary Information

Supplementary Information

Supplementary Table S1

Supplementary Table S2

Supplementary Table S3

Supplementary Table S4

## Data Availability

16S rRNA gene sequencing information has been deposited into EMBL Nucleotide Sequence Database (ENA) and can be publicly accessed at www.ebi.ac.uk/ena/data/view/PRJEB12306.
