# Supplementary material for: Low-dose exposure of glyphosate-based herbicides disrupt the urine metabolome and its interaction with gut microbiota
Source: Sci Rep. 2021 Feb 5;11:3265. doi: 10.1038/s41598-021-82552-2 (PMC7864973; doi:10.1038/s41598-021-82552-2)
Supplement: Supplementary file 1 — Supplementary Information [file 41598_2021_82552_MOESM1_ESM.docx]

**Supplementary Information**

**Low-dose exposure of glyphosate-based herbicides disrupt the urine metabolome and its interaction with gut microbiota**

**Authors:**

Jianzhong Hu^1*^, Corina Lesseur^2^, Yu Miao^2^, Fabiana Manservisi^3,4^, Simona Panzacchi^3^, Daniele Mandrioli^3,5^, Fiorella Belpoggi^3^, Jia Chen^2^, Lauren Petrick ^2,6*^

**Institutions:**

1. Department of Genetics and Genomic Sciences, Icahn School of Medicine at Mount Sinai, New York, USA
2. Department of Environmental Medicine and Public Health, Icahn School of Medicine at Mount Sinai, New York, USA
3. Cesare Maltoni Cancer Research Center (CMCRC), Ramazzini Institute (RI), Bentivoglio, Bologna, Italy
4. Department of Veterinary Medical Sciences, University of Bologna, Italy
5. Department of Agricultural Sciences, University of Bologna, Italy
6. Institute for Exposomics Research, Icahn School of Medicine at Mount Sinai, New York, NY, USA

**Address of institution:** Icahn School of Medicine at Mount Sinai

1425 Madison Avenue, New York, New York, USA

www.mssm.edu

**Address correspondence to:**

Jianzhong Hu, PhD, Department of Genetics and Genomic Sciences, Icahn School of Medicine at Mount Sinai, 1425 Madison Avenue, New York, New York, USA Tel. +12126596881 e-mail: Jianzhong.hu@mssm.edu

Lauren Petrick, PhD, Department of Environmental Medicine and Public Health, Icahn School of Medicine at Mount Sinai, 1425 Madison Avenue, New York, New York, USA Tel. +12122417351

e-mail: [lauren.petrick@mssm.edu](mailto:lauren.petrick@mssm.edu)

**Supplementary Data**

**Supplementary Figure S1.** Unsupervised PCA analysis of untargeted metabolomics profiles by exposure (control, glyphosate or roundup) in rat female and male pups.

**Supplementary Figure S2. Classification error rates for PLS-DA analysis.** Error rates are presented for the PLS-DA analysis for each subgroup.

**Supplementary Figure S3. Metabolites selected from PLS-DA analysis.** Features with VIP score > 2.0 are listed for each subgroup PLS-DA analysis.

**Supplementary Figure S4. Metabolite abundance levels selected by PLS-DA analysis among treatment groups.** The boxplots present the median and variance of the metabolites with VIP>2.0 from PLS-DA analysis with FDR-adjusted p-values<0.05 by non-parametric Wilcoxon test.

**Supplementary Figure S5. Metabolites selected by random forests method.** Top 10 metabolites sorted by their importance score are listed for each subgroup. The metabolites colored in green and labeled are those selected as biomarkers to discriminate the two comparison groups.

Supplementary Table S1. Comparison of metabolite abundances in different exposure groups in the dams. Median and IQR are provided for each metabolite. Nominal p-values were obtained from non-parametric Wilcoxon test and The VIP scores were obtained from PLS-DA analysis. P-value1 and VIP1 are for comparisons between Glyphosate and control; P-value2 and VIP2 are for comparisons between Roundup and control; P-value3 and VIP3 are for comparisons between Glyphosate and Roundup.

Supplementary Table S2. Comparison of metabolite abundances in different exposure groups in the female pups. Median and IQR are provided for each metabolite. Nominal p-values were obtained from non-parametric Wilcoxon test and The VIP scores were obtained from PLS-DA analysis. P-value1 and VIP1 are for comparisons between Glyphosate and control; P-value2 and VIP2 are for comparisons between Roundup and control; P-value3 and VIP3 are for comparisons between Glyphosate and Roundup.

Supplementary Table S3. Comparison of metabolite abundances in different exposure groups in the male pups. Median and IQR are provided for each metabolite. Nominal p-values were obtained from non-parametric Wilcoxon test and The VIP scores were obtained from PLS-DA analysis. P-value1 and VIP1 are for comparisons between Glyphosate and control; P-value2 and VIP2 are for comparisons between Roundup and control; P-value3 and VIP3 are for comparisons between Glyphosate and Roundup.

Supplementary Table S4. Feature selection using Random Forests Boruta method. For each group, the mean, median, minimum, maximum Importance score of each metabolite, number of hits normalized to number of importance source runs as well as the final decision are listed.
